# Transarterial chemoembolization plus lenvatinib with or without programmed death-1 inhibitors for patients with unresectable hepatocellular carcinoma: A propensity score matching study

**DOI:** 10.3389/fonc.2022.945915

**Published:** 2022-10-20

**Authors:** Peng Guo, Xingtao Pi, Feng Gao, Qiang Li, Duqiang Li, Wendong Feng, Wendong Cao

**Affiliations:** ^1^ Department of Interventional Therapy, Shanxi Bethune Hospital, Shanxi Academy of Medical Sciences, Tongji Shanxi Hospital, Third Hospital of Shanxi Medical University, Taiyuan, China; ^2^ Department of Interventional Therapy, Tongji Hospital, Tongji Medical College, Huazhong University of Science and Technology, Wuhan, China; ^3^ Department of Interventional Therapy, Shanxi Provincial People´s Hospital, Taiyuan, China; ^4^ Department of Interventional Therapy, Second Hospital of Shanxi Medical University, Taiyuan, China

**Keywords:** hepatocellular carcinoma, lenvatinib, TACE, PD-1, unresectable

## Abstract

**Purpose:**

We conducted a retrospective study to compare transarterial chemoembolization (TACE) plus lenvatinib plus programmed death-1 (PD-1) inhibitors with TACE plus lenvatinib in patients with unresectable hepatocellular carcinoma (HCC).

**Patients and methods:**

Patients with HCC were analyzed from January 2018 to January 2022 in three hospitals. Patients received TACE plus lenvatinib with or without PD-1 inhibitors (TACE+L+PD-1 or TACE+L, respectively). The baseline characteristics of the two groups were compared, and propensity score matching (PSM) was performed. Overall survival (OS), progression-free survival (PFS), and objective response rate (ORR) of the two groups were compared. Adverse events in the two groups were analyzed.

**Results:**

A total of 166 patients were evaluated (TACE+L+PD-1, n = 75; TACE+L, n = 91). Before PSM, OS was prolonged in the TACE+L+PD-1 group (*p* = 0.010), but PFS was similar between the two groups (*p* = 0.18). ORR was higher in the TACE+L+PD-1 group (*p* = 0.047). After PSM, estimated OS rates at 6, 12, and 24 months were 97.9%, 84.6%, and 74.1%, respectively, in the TACE+L+PD-1 group (n = 48) and 93.1%, 66.1%, and 43.4%, respectively, in the TACE+L group (n = 48). Estimated PFS rates at 3, 6, and 12 months were 81.9%, 61.8%, and 30.9%, respectively, in the TACE+L group and 95.7%, 82.1%, and 68.4%, respectively, in the TACE+L+PD-1 group. OS, PFS, and ORR were improved in the TACE+L+PD-1 group compared to the TACE+L group (*p* = 0.030; *p* = 0.027; *p* = 0.013). The safety of the TACE+L+PD-1 regimen was acceptable.

**Conclusions:**

The addition of PD-1 inhibitors to TACE+L significantly improved clinical outcomes in patients with unresectable HCC. Side effects were manageable.

## Introduction

Liver cancer is the fifth most common cancer worldwide and the second most common cause of cancer-related death (1). Hepatocellular carcinoma (HCC) comprises 80%–90% of primary liver cancers. Treatment options include surgical resection, image-guided ablation, liver transplantation, transarterial chemoembolization (TACE), sorafenib, and lenvatinib (2). Different treatments are indicated at particular clinical stages, and combination therapies are often used (2–4). Although multiple treatment options are available, the prognosis of unresectable HCC remains poor (5).

The REFLECT trial (6) disclosed that lenvatinib was non-inferior to sorafenib in prolonging overall survival (OS) in untreated advanced HCC. Furthermore, clinically meaningful improvement was observed in progression-free survival (PFS), objective response rate (ORR), and time to progression (TTP); consequently, both lenvatinib and sorafenib have emerged as first-line treatments for advanced HCC. Combination treatment with TACE and lenvatinib for advanced HCC has achieved satisfactory efficacy (7, 8).

Although the combination of programmed death-1 (PD-1) monoclonal antibodies with other modalities yields promising outcomes (9, 10), the efficacy of PD-1 inhibitors as monotherapy is unsatisfactory. In the KEYNOTE-240 trial, the addition of pembrolizumab to best supportive care as second-line therapy for advanced HCC did not improve OS and PFS per specified criteria (11). In the Check-Mate 459 trial of patients with advanced HCC, nivolumab did not improve OS and PFS when compared to sorafenib (12).

Combination therapies (including lenvatinib plus PD-1 inhibitors) have yielded promising results in patients with advanced HCC. A phase Ib study of lenvatinib plus pembrolizumab for unresectable HCC demonstrated an ORR of 46%, a median PFS of 9.3 months by modified Response Evaluation Criteria In Solid Tumors (mRECIST) criteria, and a median OS of 22 months (13). Based on the potential clinical benefits of combining lenvatinib with PD-1 inhibitors, combinations of TACE or hepatic arterial infusion chemotherapy (HAIC) with lenvatinib and PD-1 inhibitors have been studied for the treatment of advanced HCC and have demonstrated improved survival and tumor responses (14, 15).

However, the optimal combination therapy of unresectable HCC is undefined. To further evaluate the treatment of HCC, we designed a retrospective study to compare TACE plus lenvatinib plus PD-1 inhibitors (TACE+L+PD-1) with TACE plus lenvatinib (TACE+L) in patients with unresectable HCC.

## Patients and methods

### Study design and patient selection

The Ethics Board of the Shanxi Bethune Hospital, Shanxi Provincial People’s Hospital, and Second Hospital of Shanxi Medical University approved this retrospective cohort study. An informed consent requirement was waived due to the retrospective study design and anonymized data. Patients diagnosed with HCC were analyzed from January 2018 to January 2022 in Shanxi Bethune Hospital, Shanxi Provincial People’s Hospital, and the Second Hospital of Shanxi Medical University. All patients were diagnosed with HCC by non-invasive criteria or pathological examination. According to the European Association for the Study of the Liver (EASL) guidelines (16), the criteria for the non-invasive diagnosis of HCC are as follows: 1) background history of cirrhosis, 2) lesion diameter ≥1 cm, and 3) hypervascularization in the arterial phase and washout in the venous or delayed phases of four-phase multi-detector computed tomography or dynamic magnetic resonance imaging (MRI). Eligible patients were older than 18 years, in Barcelona Clinic Liver Cancer (BCLC) stages B or C (unresectable HCC), had not received previous systemic therapy, had not received previous radiotherapy or TACE, had at least one measurable lesion, had a class A Child–Pugh score, and had an Eastern Cooperative Oncology Group performance status (ECOG-PS) score of 0 to 1. Exclusion criteria were as follows: 1) previous systemic or immunotherapies, 2) follow-up interval of less than 3 months, and 3) history of autoimmune diseases.

### Baseline assessment

Baseline characteristics of age, sex, ECOG-PS, albumin–bilirubin (ALBI) grade, Child–Pugh class, tumor diameter, tumor number, BCLC stage, serum α-fetoprotein (AFP) level, presence of portal vein thrombosis, and presence of extrahepatic metastasis were compared between the two groups.

### Transarterial chemoembolization

TACE was performed by interventional radiologists with more than 5 years of experience. After a successful femoral artery puncture, a catheter (5F, RH catheter; Cook, Bloomington, IN, USA) was selected for common hepatic artery angiography. If necessary, superior mesenteric artery and phrenic artery angiography was performed. After the tumor-feeding arteries were identified, a microcatheter (SP microcatheter; Terumo, Tokyo, Japan) was used for superselective arterial embolization performed with a mixed emulsion of lipiodol (Lipiodol; Guerbet, France) and epirubicin (50 mg/m^2^). TACE was repeated every 4–6 weeks until disease progression occurred, with a limit of six TACE treatments per patient.

### Systemic therapy

Lenvatinib was administered in doses of either 12 mg (bodyweight > 60 kg) or 8 mg (bodyweight < 60 kg) orally once daily 1–3 days after TACE. PD-1 inhibitors (camrelizumab or sintilimab) were given intravenously at the standard dose (200 mg) within 7 days of the initiation of lenvatinib, with repeat dosing at 21-day intervals. PD-1 inhibitors were added to the regimens of patients who experienced tumor progression while on TACE+L treatment.

### Follow-up

Follow-up was performed every 2 months after TACE for the first three visits and every 2–3 months thereafter. Follow-up included enhanced abdominal CT or MRI and laboratory tests. Laboratory examinations included bilirubin, albumin, prothrombin time, and serum AFP levels. Adverse events were analyzed based on the National Cancer Institute Common Terminology Criteria for Adverse Events, version 4.0 (17).

### Definitions

OS was defined as the time interval from the first TACE until death or loss to follow-up. PFS was defined as the time interval from the first TACE to disease progression or loss to follow-up. Tumor progression was defined as either an increase in tumor diameter of 25% from baseline, the appearance of new lesions, macrovascular invasion, or extrahepatic metastasis (18). Complete response (CR), partial response (PR), progressive disease (PD), and stable disease (SD) assessments were performed 1 month after the first TACE. ORR included CR and PR. PFS, CR, PR, SD, and PD were estimated by mRECIST criteria.

### Statistical analysis

Categorical data are reported as number with percentage. Propensity score matching (PSM) was used to correct potential confounding biases between the two groups (a nearest-neighbor algorithm [1:1] was used to account for pre-treatment laboratory tests, imaging, and clinical covariates). The *x* ^2^ test or Fisher’s exact test was used to compare categorical data before PSM. The Cochran–Mantel–Haenszel test was used to evaluate differences in the categorical data after PSM. Primary endpoints were OS; secondary endpoints were PFS and ORR. The Kaplan–Meier curves were used to estimate OS and PFS. A comparison of survivals between the two groups was performed by using the log-rank test. All tests of significance were two-sided, and a *p*-value <0.05 was considered statistically significant. All statistical analyses were performed using EmpowerStats (www.empowerstats.com) and R software, version 3.6.2.

## Results

### Patient demographics

**Figure 1:**
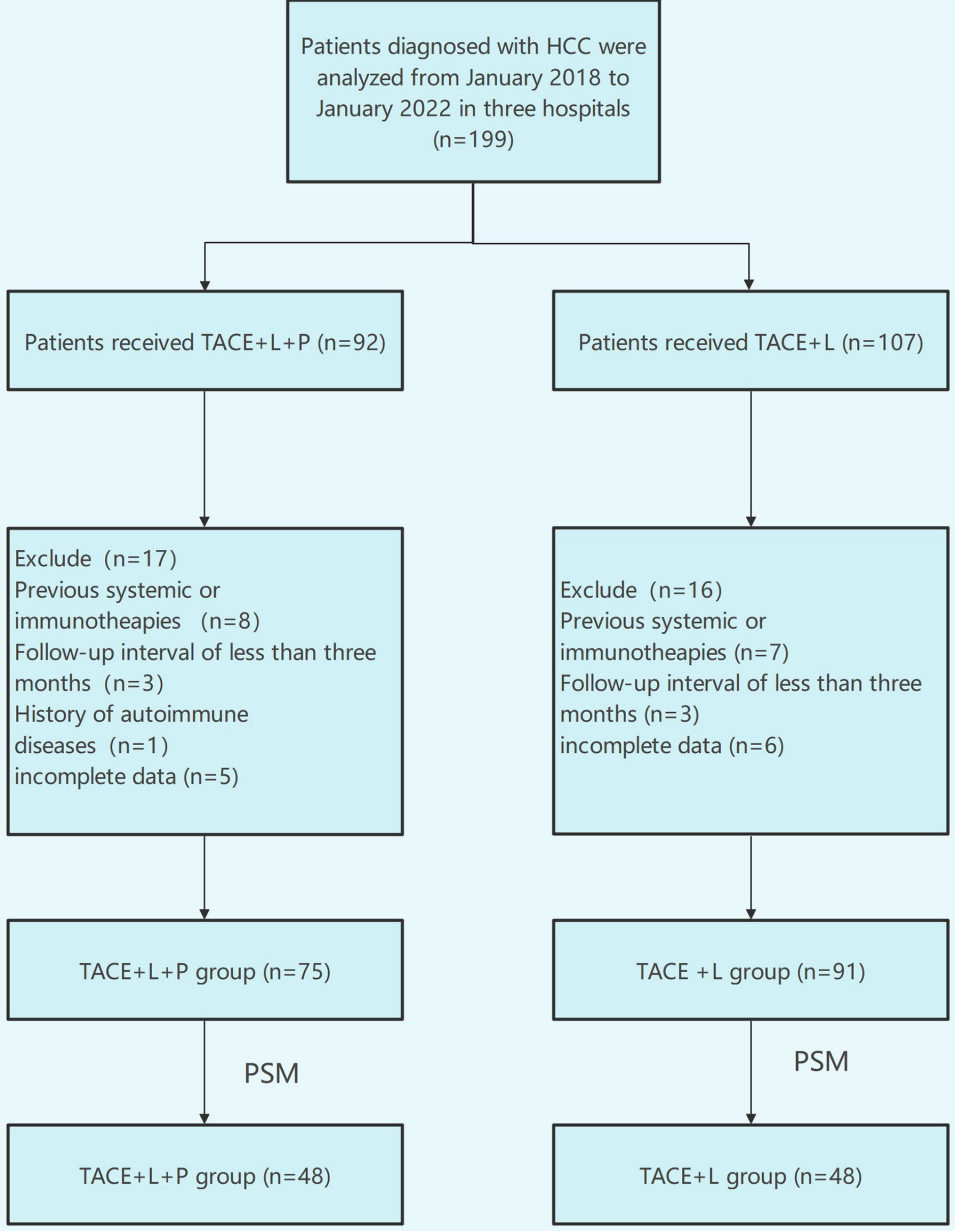
Flow diagram of patient enrollment.

A total of 199 patients were evaluated for eligibility, comprising 107 patients in the TACE+L group and 92 patients in the TACE+L+PD-1 group. A total of 33 patients were excluded because they met the exclusion criteria, and 166 patients were analyzed in this study last (**Figure 1**). Before PSM, sex, ECOG-PS, and Child–Pugh class were different between the two groups (*p* < 0.05). There were no significant differences in other indicators. After PSM, there were no significant intergroup differences in any of the baseline characteristics (**Table 1**).

**Table 1:** Baseline characteristics before and after PSM.

Variables	Before PSM			After PSM		
	TACE+L n=91n, (%)	TACE+L+PD1 n=75n, (%)	*P*	TACE+L n=48n, (%)	TACE+L+PD1 n=48n, (%)	*P*
Age			0.263			0.593
≤60	66 (72.5)	60 (80.0)		41 (85.4)	38 (79.2)	
>60	25 (27.5)	15 (20.0)		7 (14.6)	10 (20.8)	
Sex			0.017			1.000
Female	3 (3.3)	10 (13.3)		2 (4.2)	1 (2.1)	
Male	88 (96.7)	65 (86.7)		46 (95.8)	47 (97.9)	
ECOG-PS			<0.001			1.000
0	58 (63.7)	69 (92.0)		43 (89.6)	43 (89.6)	
1	33 (36.3)	6 (8.0)		5 (10.4)	5 (10.4)	
ALBI grade			0.292			0.320
1	34 (37.4)	37 (49.3)		20 (41.7)	25 (52.1)	
2	55 (60.4)	37 (49.3)		28 (58.3)	22 (45.8)	
3	2 (2.2)	1 (1.3)		0 (0)	1 (2.1)	
Child-Pugh Class			0.009			0.673
A	78 (85.7)	73 (97.3)		44 (91.7)	46 (95.8)	
B	13 (14.3)	2 (2.7)		4 (8.3)	2 (4.2)	
Tumor diameter (cm)			0.863			0.527
≤7	34 (37.4)	29 (38.7)		20 (41.7)	16 (33.3)	
>7	57 (62.6)	46 (61.3)		28 (58.3)	32 (66.7)	
Tumor number (n)			0.697			0.173
≤3	28 (30.8)	21 (28.0)		10 (20.8)	17 (35.4)	
>3	63 (69.2)	54 (72.0)		38 (79.2)	31 (64.6)	
BCLC stage			0.727			0.511
B	20(22.0)	20 (26.7)		12 (25.0)	9 (28.8)	
C	71(78.0)	55 (73.3)		36 (75.0)	39 (81.2)	
AFP (ng/ml)			0.086			0.674
≤400	41 (45.1)	24 (32.0)		20 (41.7)	17 (35.4)	
>400	50 (54.9)	51 (68.0)		28 (58.3)	31 (64.6)	
Portal vein thrombosis			0.622			0.671
NO	33 (36.3)	30 (40.0)		19 (39.6)	16 (33.3)	
Yes	58 (63.7)	45 (60.0)		29 (60.4)	32 (66.7)	
Extrahepatic metastasis			0.207			0.538
NO	42 (46.2)	42 (56.0)		28 (58.3)	24 (50)	
Yes	49 (53.8)	33 (44.0)		20 (41.7)	24 (50)	

ALBI, albumin-bilirubin; AFP, alpha-fetoprotein; BCLC, Barcelona Clinic Liver Cancer.

### Overall survival

**Figure 2:**
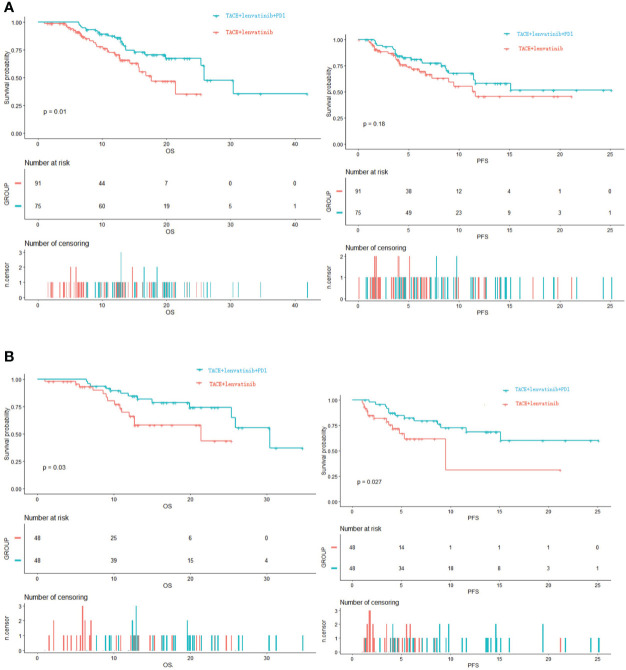
**(A)** Kapan–Meier curves of OS and PFS before PSM. **(B)** Kapan–Meier curves of OS and PFS after PSM. OS, overall survival; PFS, progression-free survival; PSM, propensity score matching.

The median follow-up time was 14.7 months (95% CI: 12.2–17.1). Before PSM, the median OS in the TACE+L group was 17.6 months (95% CI: 12.9-22.3); estimated OS rates at 6, 12, and 24 months were 91.3%, 70.4%, and 35.1%, respectively. In the TACE+L+PD-1 group, the median OS was 25.9 months (95% CI: 21.8–30.0); estimated OS rates at 6, 12, and 24 months were 93.3%, 89.1%, and 61.0%, respectively. The Kaplan–Meier curves showed significant intergroup differences in OS rates (*p* = 0.010) (**Figure 2A**).

After PSM, the median OS was 21.4 months (95% CI: 12.7–not estimable) in the TACE+L group; estimated OS rates at 6, 12, and 24 months were 93.1%, 66.1%, and 43.4%, respectively. The median OS was 30.4 months (95% CI: 25.4–not estimable) in the TACE+L+PD-1 group; estimated OS rates at 6, 12, and 24 months were 97.9%, 84.6%, and 74.1%, respectively. The Kaplan–Meier curves showed significant intergroup differences in OS (*p* = 0.030, **Figure 2B**).

### Progression-free survival

Before PSM, the estimated PFS rates in the TACE+L group at 3, 6, and 12 months were 86.8%, 69.3%, and 45.5%, respectively, and 91.6%, 79.1%, and 57.9%, respectively, in the TACE+L+PD-1 group. Although the PFS rates of the TACE+L+PD-1 group were higher than those of the TACE+L group, the Kaplan–Meier curves were similar (*p* = 0.18, **Figure 2A**).

Estimated PFS rates after PSM at 3, 6, and 12 months were 81.9%, 61.8%, and 30.9%, respectively, in the TACE+L group. In contrast, estimated PFS rates at the corresponding time points were 95.7%, 82.1%, and 68.4%, respectively, in the TACE+L+PD-1 group. The Kaplan–Meier curves showed significant intergroup differences in PFS (*p* = 0.027, **Figure 2B**).

### Objective response rate

In the TACE+L group, 3, 45, 20, and 12 patients developed CR, PR, SD, and PD, respectively. Four, 47, 11, and 23 patients in the TACE+L+PD-1 group developed CR, PR, SD, and PD, respectively. The ORR before PSM was 52.7% in the TACE+L group and 68.0% in the TACE+L+PD-1 group. The ORRs of the two groups were significantly different (*p* = 0.047) (**Table 2**).

**Table 2:** Tumor response before and after PSM.

	Before PSM			After PSM		
	TACE+L n=91n, (%)	TACE+L+PD-1 n=75n, (%)	*P*	TACE+L n=48n, (%)	TACE+L+PD-1 n=48n, (%)	*P*
CR	3 (3.3)	4 (5.3)		1 (2.1)	2 (4.2)	
PR	45 (49.5)	47 (62.7)		18 (37.5)	31 (64.6)	
SD	20 (22.0)	11 (14.7)		12 (25.0)	7 (14.6)	
PD	23 (25.3)	13 (17.3)		17 (35.4)	8 (16.7)	
ORR	48 (52.8)	51 (68.0)	0.046	19 (39.6)	33 (66.8)	0.013

PSM, propensity score matching; TACE, transarterial chemoembolization, L, lenvatinib; PD-1, anti-PD-1 blockade; CR, complete response; PR, partial response; SD, stable disease; PD, progressive disease; ORR, overall response rate.

After PSM, 1, 18, 12, and 17 patients in the TACE+L group experienced CR, PR, SD, and PD, respectively, while 2, 31, 7, and 8 patients in the TACE+L+PD-1 group developed CR, PR, SD, and PD, respectively. ORRs were 41.7% and 68.8% in the TACE+L and TACE+L+PD-1 groups, respectively, and were significantly different (*p* = 0.013).

### Adverse events

Forty-five (49.5%) and 62 (82.7%) patients reported adverse events (AEs) of any grade in the TACE+L and TACE+L+PD-1 groups, respectively (**Table 3**). Fatigue, hypertension, diarrhea, and decreased appetite were the most common AEs. Seven patients (7.7%) experienced grade 3 or higher AEs in the TACE+L group, while 23 patients (30.7%) developed grade 3 or higher AEs in the TACE+L+PD-1 group. Thirty-three (44.0%) and 9 (12.0%) patients in the TACE+lenvatinib+PD-1 group required dose reductions or discontinuations, respectively, because of treatment-related AEs. Treatments were reduced in 11 (12.1%) and discontinued in 2 (2.1%) patients in the TACE+L group because of treatment-related AEs. No treatment-related deaths occurred in either group.

**Table 3:** Treatment-related adverse events.

AEs	TACE+L n=91n, (%)		TACE+L+PD-1 n=75n, (%)	
	Any Grade	≥ Grade 3	Any Grade	≥ Grade 3
Hypertension	11 (12.1)	3 (3.3)	19 (25.3)	6 (8.0)
Fatigue	10 (10.1)	2 (2.2)	10 (13.3)	4 (5.3)
Diarrhea	7 (7.7)	0 (0)	7 (9.3)	4 (5.3)
Decreased appetite	8 (8.8)	2 (2.2)	8 (10.7)	3 (4.0)
Nausea	7 (7.7)	0 (0)	7 (9.3)	3 (4.0)
Palmar-plantar Erythrodysesthesia syndrome	1 (1.1)	0 (0)	4 (5.3)	1 (1.3)
Proteinuria	0 (0)	0 (0)	1 (1.3)	0 (0)
Abnormal liver function	0 (0)	0 (0)	2 (2.6)	1 (1.3)
Thrombocytopenia	0 (0)	0 (0)	1 (1.3)	0 (0)
Abdominal pain	0 (0)	0 (0)	1 (1.3)	1 (1.3)
Hypothyroidism	0 (0)	0 (0)	1 (1.3)	0 (0)
Rash	1 (1.1)	0 (0)	1 (1.3)	0 (0)

TACE, transarterial chemoembolization; PD-1, anti-PD-1 blockade.

## Discussion

TACE is the first-line treatment for mid-stage HCC according to the EASL guidelines and the BCLC treatment strategy. However, the efficacy of TACE monotherapy of unresectable and advanced HCC is unsatisfactory. In contrast, the combination of TACE with lenvatinib has yielded favorable results (8, 19, 20) and is better than TACE monotherapy. In the LAUNCH trial, a phase III and randomized clinical trial, TACE combined with lenvatinib *vs.* lenvatinib alone as first-line treatment for advanced hepatocellular carcinoma, the median OS and PFS were significantly longer in the TACE+L group than lenvatinib group (17.8 *vs.* 11.5 months; 10.6 *vs.* 6.4 months); patients in the TACE+L group had a significantly higher ORR according to the modified RECIST criteria (54.1% *vs.* 25.0%) (21). Furthermore, triple therapy consisting of TACE, lenvatinib, and PD-1 inhibitors has improved clinical outcomes while causing controllable side effects (7, 22).

In our study, our findings of higher OS and ORR before PSM and improved OS, PFS, and ORR after PSM in the TACE+L+PD-1 group compared to TACE+L recipients suggest that the addition of PD-1 inhibitors conferred significant survival benefits. The efficacy of triple therapy in our study was better than or similar to previously reported results. In a prospective cohort study of TACE combined with lenvatinib plus PD-1 inhibitors compared with TACE alone, the median OS was 23.9 months and the ORR was 67.9% in the TACE+L+PD-1 group (22). Another study of TACE combined with lenvatinib plus PD-1 inhibition for advanced HCC reported a median OS of 16.9 months and an ORR of 56.1% (23).

The majority of our patients presented with more than three tumors, which may suggest that TACE+L+PD-1 triple therapy improves the outcomes of multifocal HCC. The high prevalence of BCLC stage C (portal vein thrombosis and/or extrahepatic metastases) in our study population (**Table 1**) may also indicate that patients with portal vein thrombosis or extrahepatic metastasis derive further clinical benefit from the addition of PD-1 inhibitors to TACE combined with lenvatinib.

The safety of TACE+lenvatinib+PD-1 was also acceptable. Although dose reductions and treatment discontinuations due to treatment-related AEs were indicated in 44.0% and 12.0%, respectively, in the TACE+L+PD-1 group, there were no treatment-related deaths.

Our study has several limitations. The first is its retrospective design, which may lead to selection bias. Second, because of the limited number of cases, no subgroup analyses were performed in either treatment group. After PSM, each group included a relatively small sample size of 48 patients. Consequently, our findings should be confirmed by a large-sample and randomized controlled study.

## Conclusion

For unresectable HCC, TACE combined with lenvatinib and PD-1 inhibitors was associated with significantly better OS, PFS, and ORR than TACE combined with lenvatinib. Side effects were manageable.

## Data availability statement

The raw data supporting the conclusions of this article will be made available by the authors, without undue reservation.

## Ethics statement

This study was reviewed and approved by third hospital of Shanxi medical university, Shanxi provincial people´s hospital and second hospital of Shanxi medical university. The patients/participants provided their written informed consent to participate in this study.

## Author contributions

PG: paper writing,data collection and design. FG: data collection and follow-up. QL: data collection and follow-up. DL: data collection. WF: data collection. WC: paper writing and design. All authors contributed to the article and approved the submitted version.

## Funding

This work has been financially supported by the foundation of ShanXi Academy of Medical Sciences (No. REMD03).

## Conflict of interest

The authors declare that the research was conducted in the absence of any commercial or financial relationships that could be construed as a potential conflict of interest.

## Publisher’s note

All claims expressed in this article are solely those of the authors and do not necessarily represent those of their affiliated organizations, or those of the publisher, the editors and the reviewers. Any product that may be evaluated in this article, or claim that may be made by its manufacturer, is not guaranteed or endorsed by the publisher.
